# Prevalence of metal implants among US adults aged 40 years and older

**DOI:** 10.1038/s41598-024-84340-0

**Published:** 2025-01-02

**Authors:** Qiu-Fu Wang, Yu-Chen Tang, Hao-Ran Liao, Miao Lei, Wei Dong, Ze-Yu Liu, Jie Hao, Zhen-Ming Hu

**Affiliations:** 1 Present Address: Department of Orthopedics, University-Town Hospital of Chongqing Medical University, Chongqing, China; 2 Present Address: Department of Orthopedics, The First Affiliated Hospital of Chongqing Medical University, Chongqing, China; 3 Department of Spinal Surgery, Chongqing Orthopedic Hospital of Traditional Chinese Medicine, Chongqing, China

**Keywords:** Metal implants, Orthopaedics, Epidemiology, Public health, Epidemiology, Epidemiology

## Abstract

Metal implants are commonly used in clinical practice. However, little is known regarding the prevalence of metal implants. Therefore, this study aimed to evaluate the prevalence of metal implants in the United States (US) among individuals aged ≥ 40 years. This study conducted a serial cross-sectional analysis of US adults aged ≥ 40 years who participated in the National Health and Nutrition Examination Survey (NHANES) (2015–2016 and 2017–March 2020). Self-reported questionnaires were used to assess whether the participants had metal implants inside their bodies. The primary outcome was the prevalence of metal implants among adults aged 40 years and older. Furthermore, weighted logistic regression analysis was employed to determine the changes in the prevalence of metal implants from 2015 to March 2020. Moreover, this study investigated the variation in metal implant prevalence by demographic factors based on the pooled NHANES cycles. All analyses were conducted based on 3,736 participants from the NHANES 2015–2016 and 6,387 participants from the NHANES 2017–March 2020. This study observed a high prevalence of metal implants among adults aged 40 and older (2015–2016: 27.23%; 2017–March 2020: 31.53%). Moreover, the results of the weighted logistic regression analysis showed that the prevalence of metal implants significantly increased from 2015 to March 2020, especially among older individuals, men, and White individuals. In addition, the results of the weighted logistic regression analysis indicated that the metal implant prevalence differed by age and race/ethnicity, in which older individuals and White individuals showed a significantly higher prevalence of metal implants than younger individuals and non-White individuals, respectively. There was a high prevalence of metal implants among US adults aged 40 and older, and the prevalence of metal implants significantly increased from 2015 to March 2020. Therefore, more attention needs to be paid to this special population, and it may be necessary to ensure accessibility and affordability and assess the potential long-term health impacts of metal implants, considering the increased prevalence of metal implants.

**Supplementary Information:**

The online version contains supplementary material available at 10.1038/s41598-024-84340-0.

## Introduction

Metal implants, medical devices made from biocompatible metals, are commonly used in clinical settings, with about 70% of Food and Drug Administration (FDA) approved medical implants that are metal‐based^1^. Metal implants, typically made from materials such as titanium, stainless steel, cobalt-chromium alloys, or a combination of these metals, are commonly used to replace damaged or diseased bones or joints, providing stability, support, and restoring functionality to the affected area^2,3^. Furthermore, metal implants have been widely used in the field of orthopaedics, such as joint replacements, fracture fixation, spinal fusion, and bone reconstruction^4–7^. Take joint replacements as an example. Metal implants, which are considered the ideal material for replacing diseased bone and joint tissues, are used extensively in joint replacement surgery, such as total hip arthroplasty (THA) and total knee arthroplasty (TKA)^8–11^. One study reports that the annual procedure volumes of primary THA and primary TKA in the United States (US) in 2014 were about 370,770 and 680,150, respectively^12^. In addition, a large number of studies suggest that the use of THA and TKA is expected to increase significantly over time^12–18^. Although the above evidence is unable to reflect the epidemiology of metal implants directly and only reveals the tip of an iceberg, it is not hard to see that there is a large population of individuals with metal implants, especially with the increase in the aging population.

Although it is well recognized that metal implants play important roles in improving clinical outcomes, cumulative evidence indicates that metal implants may cause potential short- and long-term negative impacts on human health, such as infection, implant loosening, metal hypersensitivity, elevated concentrations of metal ions, wear and tear over time, and the possibility of requiring revision surgeries^19–25^. Furthermore, considering the efficacy and safety of the existing metal implants, the design and development of novel, more efficient, and safer metal implants has recently become an important research field and hot spot^1,26–28^. The above evidence suggests that the issues associated with metal implants have been widely discussed and received growing attention. Therefore, it is necessary to pay more attention to this special population, individuals with metal implants inside the body, and evaluate the prevalence of metal implants, which might play an essential role in supporting evidence-based decision-making, facilitating related research advancements, enhancing implant safety, and developing effective integrated management strategies in the future. However, to the best of our knowledge, none has concentrated on the prevalence of subjects with metal implants.

Against this background, this study aimed to evaluate the prevalence of metal implants among adults aged 40 and older in the United States (US) using the data from the National Health and Nutrition Examination Survey (NHANES). Moreover, this study evaluated the changing trends in the prevalence of metal implants from 2015 to March 2020. In addition, this study investigated whether the prevalence of metal implants varied by age, sex, and race/ethnicity. These exploratory attempts mentioned above might aid the understanding of the current status of the use of metal implants, which could help guide clinical strategies and public health policies.

## Material and methods

### Study design and populations

This serial cross-sectional study included participants aged 40 years and older (questions on metal implants were asked of only survey participants 40 years and older) from the NHANES (2015–2016 and 2017-March 2020), considering the questionnaire on metal implants was not evaluated before NHANES 2015–2016. Furthermore, this study excluded subjects without definitive answers to the question on metal implants (refused to answer or answered “don’t know.”) or with missing data on metal implants. The NHANES, which was conducted by the National Center for Health Statistics (NCHS) of the Centers for Disease Control and Prevention (CDC), was designed to evaluate the health and nutritional status of US residents. Moreover, all participants from the NHANES have received and signed the informed consent, and the ethics review board of the NCHS has approved the NHANES^29–31^. Additional information was provided at the NHANES website (https://www.cdc.gov/nchs/nhanes/index.htm). This study has been reported in line with the Strengthening the Reporting of Observational Studies in Epidemiology (STROBE) reporting guideline^32^.

### Questions on metal implants

A self-reported questionnaire, in which participants were asked the question “any metal objects inside your body,” was used to evaluate whether subjects were with metal implants. The definition of metal implants in the question contained any artificial joints, pins, plates, metal suture material, or other types of metal objects in the body, and the metal object should not be visible on the outside of the body or in the mouth (such as piercings, crowns, dental braces or retainers, shrapnel, or bullets). Other details about the questionnaire on metal implants are provided on NHANES^33–35^.

### Statistical analysis

All analyses were performed from December 1, 2023, to January 5, 2024. This study first described the demographic characteristics and medical history (age, sex, race/ethnicity and diseases that led to the placement of the implants, such as arthritis and coronary heart disease) of participants included in the final analysis, in which continuous variables were reported as means and 95% confidence intervals (CIs, using Wald type confidence interval), while categorical variables were reported as percentages with 95% CIs. For demographic characteristics and medical history, missing data (participants who (i) refused to answer the question, (ii) answered “don’t know.” to the question, or (iii) with missing data) were reported as unknown. Furthermore, differences in demographic characteristics between NHANES 2015–2016 and NHANES 2017-March 2020 for continuous variables were assessed using survey-weighted linear regression, whereas categorical variables were compared using survey-weighted Chi-square test. Moreover, this study estimated the overall prevalence of metal implants and prevalence stratified by demographic factors in survey cycles 2015–2016 and 2017-March 2020. In addition, weighted logistic regression models were employed to assess the change in the prevalence of metal implants from survey cycles 2015–2016 to 2017-March 2020. In addition, the associations between demographic factors and the prevalence of metal implants were investigated based on the pooled NHANES cycles (2015–2016 and 2017-March 2020) using the weighted logistic regression models. In sensitivity analysis, this study assessed the prevalence of metal implants after excluding participants with missing data on demographic characteristics and medical history. Nationally representative estimates were calculated for all analyses by utilizing NHANES survey weights, which effectively accommodate the intricate, cluster-stratified design of NHANES^36^. Statistics were generated using R software version 4.2.1 (https://cran.r-project.org/) and EmpowerStats version 2.0 (http://www.empowerstats.com). A result was considered significant when its 2-sided P value was less than 0.05.

## Results

### Baseline characteristics

**Fig. 1:**
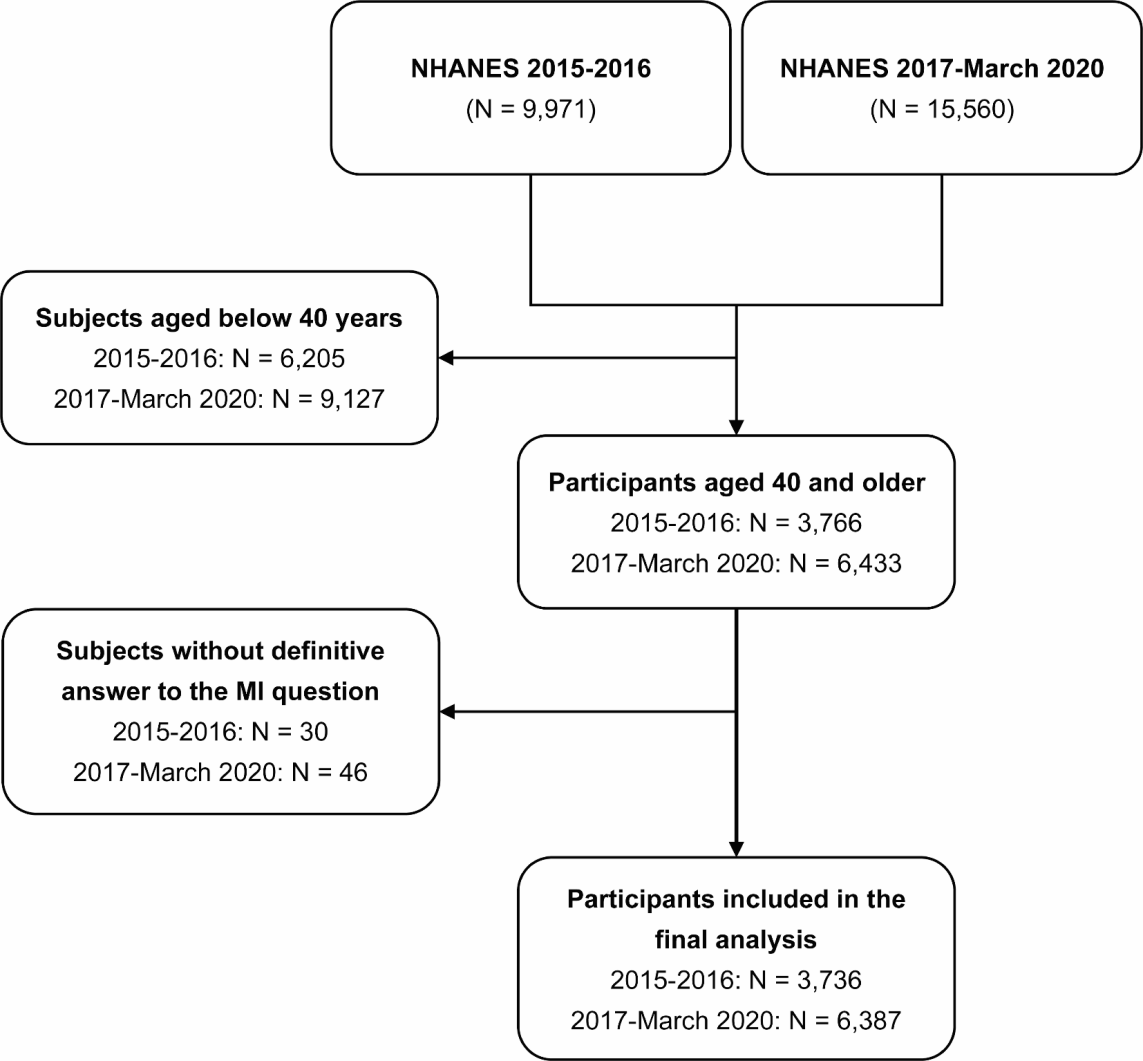
Flowchart of participant selection. MI, metal implant; NHANES, National Health and Nutrition Examination Surveys.

The flowchart of participant selection is shown in Fig. 1. For subjects aged 40 years and older, 74 participants (NHANES 2015–2016: N = 28; NHANES 2017-March 2020: N = 46) who answered “don’t know” to the questions on metal implants and two participants with missing data on metal implants (all from the NHANES 2015–2016) were excluded from the present study. Finally, a total of 10,123 participants from the NHANES (2015–2016: N = 3,736; 2017-March 2020: N = 6,387) were included in the final analysis. Moreover, the mean ages of participants from the NHANES 2015–2016 and NHANES 2017-March 2020 were 58.47 (95% CI: 57.56 to 59.37) and 59.05 (95% CI: 58.34 to 59.77), respectively. No significant differences in demographic characteristics except for the history of stroke (2015–2016: 4.02% vs. 2017-March 2020: 5.58%) between participants from NHANES 2015–2016 and NHANES 2017-March 2020 were observed. Other details on demographic information are listed in Table 1. In addition, there were no differences in age, sex, and race/ethnicity between included and excluded participants aged 40 and older (other details on demographic information are shown in Table 2).

**Table 1:** Demographic factors of participants included in the final analysis.

Demographic factor	NHANES 2015–2016	NHANES 2017–March 2020	*P* value ^b^
	(N = 3,736)^a^	(N = 6,387)^a^	
Age (years), mean^c^	58.47 (57.56, 59.37)	59.05 (58.34, 59.77)	0.326
Sex, %^c^			0.934
Men	47.20 (45.52, 48.90)	47.30 (45.69, 48.91)	
Women	52.80 (51.10, 54.48)	52.70 (51.09, 54.31)	
Race/ethnicity, %^c^			0.873
White	68.68 (60.27, 76.02)	66.76 (61.71, 71.45)	
Black	10.56 (6.97, 15.70)	10.66 (8.11, 13.88)	
Mexican American	6.92 (3.98, 11.77)	6.48 (4.92, 8.49)	
Other	13.84 (10.22, 18.48)	16.10 (13.51, 19.08)	
History of arthritis, %			0.768
Yes	38.20 (35.84, 40.56)	39.18 (36.30, 42.05)	
No	61.58 (59.24, 63.93)	60.61 (57.73, 63.48)	
Unknown^d^	0.22 (0.03, 0.41)	0.22 (0.14, 0.30)	
History of congestive heart failure, %			0.969
Yes	3.77 (3.15, 4.39)	3.87 (3.06, 4.67)	
No	95.95 (95.27, 96.64)	95.88 (95.06, 96.71)	
Unknown^d^	0.28 (− 0.02, 0.57)	0.25 (0.10, 0.40)	
History of coronary heart disease, %			0.267
Yes	5.34 (4.34, 6.35)	6.31 (4.69, 7.93)	
No	94.24 (93.18, 95.30)	93.47 (91.83, 95.11)	
Unknown^d^	0.42 (0.13, 0.70)	0.22 (0.11, 0.33)	
History of angina/angina pectoris, %			0.468
Yes	2.99 (2.35, 3.63)	3.28 (2.62, 3.94)	
No	96.81 (96.15, 97.48)	96.41 (95.71, 97.11)	
Unknown^d^	0.20 (0.06, 0.33)	0.31 (0.22, 0.41)	
History of heart attack, %			0.586
Yes	5.07 (4.21, 5.93)	5.56 (4.37, 6.76)	
No	94.84 (93.99, 95.68)	94.28 (93.08, 95.49)	
Unknown^d^	0.09 (0.00, 0.18)	0.15 (0.02, 0.29)	
History of stroke, %			0.018
Yes	4.02 (3.47, 4.57)	5.58 (4.82, 6.34)	
No	95.88 (95.26, 96.50)	94.30 (93.52, 95.07)	
Unknown^d^	0.10 (− 0.04, 0.25)	0.13(0.05, 0.20)	
History of cancer, %			0.221
Yes	16.20 (15.12, 17.28)	16.60 (15.20, 17.99)	
No	83.69 (82.56, 84.83)	83.38 (82.00, 84.77)	
Unknown^d^	0.11 (− 0.07, 0.29)	0.02 (0.00, 0.04)	

^a^Unweighted sample size.

^b^*P* value determined by survey-weighted linear regression (for continuous variables) or survey-weighted Chi-square test (for categorical variables).

^c^Weighted mean or percentage.

^d^Participants who (i) refused to answer the question, (ii) answered “don’t know.” to the question, or (iii) with missing data.

NHANES, National Health and Nutrition Examination Survey.

**Table 2:** Demographic factors between included and excluded participants.

Demographic factor	Included	Excluded^a^	*P* value^c^
	(N = 10,123)^b^	(N = 76)^b^	
Age (years), mean	58.83 (58.27, 59.39)	62.36 (58.01, 66.71)	0.117
Sex, %			0.518
Men	47.26 (46.11, 48.42)	41.15 (23.5, 61.42)	
Women	52.74 (51.58, 53.89)	58.85 (38.58, 76.50)	
Race/ethnicity, %			0.718
White	67.49 (63.22, 71.48)	71.71 (56.2, 83.35)	
Black	10.62 (8.5, 13.19)	7.19 (3.43, 14.45)	
Mexican American	6.65 (5.11, 8.60)	5.90 (2.44, 13.59)	
Other	15.25 (13.12, 17.65)	15.21 (7.76, 27.65)	
History of arthritis, %			0.032
Yes	38.81 (36.81, 40.81)	42.39 (26.34, 58.43)	
No	60.98 (58.98, 62.97)	55.45 (39.11, 71.79)	
Unknown^d^	0.22 (0.13, 0.30)	2.16 (− 2.06, 6.39)	
History of congestive heart failure, %			0.029
Yes	3.83 (3.28, 4.38)	8.31 (− 0.01, 16.63)	
No	95.91 (95.34, 96.48)	89.82 (80.90, 98.75)	
Unknown^d^	0.26 (0.12, 0.40)	1.87 (− 1.29, 5.02)	
History of coronary heart disease, %			0.008
Yes	5.94 (4.86, 7.03)	12.96 (2.26, 23.66)	
No	93.76 (92.66, 94.86)	84.61 (73.16, 96.06)	
Unknown^d^	0.30 (0.17, 0.42)	2.43 (− 1.83, 6.70)	
History of angina/angina pectoris, %			< 0.001
Yes	3.17 (2.69, 3.65)	0.68 (− 0.26, 1.62)	
No	96.56 (96.05, 97.07)	91.28 (86.43, 96.12)	
Unknown^d^	0.27 (0.19, 0.35)	8.04 (3.33, 12.75)	
History of heart attack, %			< 0.001
Yes	5.38 (4.56, 6.19)	3.74 (− 1.89, 9.38)	
No	94.49 (93.68, 95.31)	90.03 (80.85, 99.20)	
Unknown^d^	0.13 (0.04, 0.22)	6.23 (− 1.18, 13.64)	
History of stroke, %			< 0.001
Yes	4.99 (4.47, 5.50)	4.31 (− 0.52, 9.15)	
No	94.90 (94.36, 95.43)	90.57 (86.08, 95.06)	
Unknown^d^	0.12 (0.04, 0.19)	5.12 (− 2.08, 12.32)	
History of cancer, %			< 0.001
Yes	16.45 (15.49, 17.40)	23.47 (12.00, 34.93)	
No	83.50 (82.54, 84.47)	74.37 (62.42, 86.32)	
Unknown^d^	0.05 (− 0.02, 0.12)	2.16 (− 2.06, 6.39)	

% Weighted percentage.

^a^Subject aged 40 and older without definitive answer to the question on metal implants (refused to answer or answered “don’t know”) or with missing data on metal implants.

^b^Unweighted sample size.

^c^P value determined by survey-weighted linear regression (for continuous variables) or survey-weighted Chi-square test (for categorical variables).

^d^Participants who (i) refused to answer the question, (ii) answered “don’t know.” to the question, or (iii) with missing data.

### Prevalence of metal implants among individuals aged 40 and older

Overall, the prevalence of metal implants among those 40 years and older in 2015–2016 and 2017-March 2020 were 27.23% (95% CI: 23.61 to 30.85) and 31.53% (95% CI: 29.76 to 33.30), respectively. Furthermore, the prevalence of metal implants showed an increasing trend from 2015 to March 2020, especially for the older population (60 years and older) (from 37.19% to 43.40%), men (from 25.89% to 32.05%), Whites (from 29.76% to 36.00%), and Mexican American (from 19.07% to 23.64%). Moreover, the older population and Whites, regardless of survey cycles, showed a higher prevalence of metal implants than the younger population (40–59 years) and non-Whites, respectively. Results are listed in Table 3.

**Table 3:** Prevalence of metal implants among US adults 40 years or older.

	NHANES 2015–2016		NHANES 2017–March 2020		
Population	Unweighted sample size	Prevalence % (95% CI)	Unweighted sample size	Prevalence % (95% CI)	P-value
Overall	3736	27.23 (23.61, 30.85)	6387	31.53 (29.76, 33.30)	0.049
Age					
40–59 years	1853	19.50 (15.69, 23.31)	2993	21.21 (19.06, 23.37)	0.456
60 years and older	1883	37.19 (32.77, 41.60)	3394	43.40 (41.04, 45.76)	0.022
Sex					
Men	1796	25.89 (22.04, 29.74)	3154	32.05 (29.33, 34.78)	0.017
Women	1940	28.43 (23.84, 33.02)	3233	31.06 (28.96, 33.16)	0.322
Race/ethnicity					
White	1277	29.76 (25.46, 34.05)	2381	36.00 (33.06, 38.94)	0.027
Black	795	20.71 (18.02, 23.39)	1710	20.54 (18.34, 22.74)	0.926
Mexican American	622	19.07 (16.69, 21.45)	647	23.64 (21.36, 25.93)	0.011
Other	1042	23.76 (18.32, 29.19)	1649	23.46 (20.01, 26.90)	0.927

CI, confidence interval; NHANES, National Health and Nutrition Examination Survey.

### Change in metal implant prevalence from 2015 to March 2020

The results of weighted logistic regression analysis showed that the overall prevalence of metal implants significantly increased from 2015 to March 2020, regardless of adjustment for age, sex, and race/ethnicity. In subgroup analysis, this study found that the prevalence of metal implants significantly increased among the older population (60 years and older), men, and Whites from 2015 to March 2020, regardless of adjustment for demographic factors. However, no significant increase in the prevalence of metal implants was observed among the younger population (40–59 years), women, and non-Whites from 2015 to March 2020. Other details are listed in Table 4.

**Table 4:** Change in prevalence of metal implants.

Population	Survey cycle	Model 1^a^		Model 2^b^	
		OR (95% CI)	*P* value	OR (95% CI)	*P* value
Overall	2015–2016	Reference (1)	–	Reference (1)	–
	2017–March 2020	1.23 (1.01, 1.50)	0.049	1.22 (1.01, 1.48)	0.043
Age^c^					
40–59 years	2015–2016	Reference (1)	–	Reference (1)	–
	2017–March 2020	1.11 (0.84, 1.46)	0.456	1.12 (0.85, 1.48)	0.433
60 years and older	2015–2016	Reference (1)	–	Reference (1)	–
	2017–March 2020	1.30 (1.05, 1.60)	0.022	1.31 (1.07, 1.62)	0.015
Sex^d^					
Men	2015–2016	Reference (1)	–	Reference (1)	–
	2017–March 2020	1.35 (1.07, 1.71)	0.017	1.35 (1.09, 1.69)	0.011
Women	2015–2016	Reference (1)	–	Reference (1)	–
	2017–March 2020	1.13 (0.89, 1.45)	0.322	1.12 (0.87, 1.44)	0.377
Race/ethnicity^e^					
White	2015–2016	Reference (1)	–	Reference (1)	–
	2017–March 2020	1.33 (1.04, 1.69)	0.027	1.30 (1.03, 1.65)	0.033
Non-White	2015–2016	Reference (1)	–	Reference (1)	–
	2017–March 2020	1.05 (0.86, 1.29)	0.633	1.04 (0.84, 1.28)	0.740

^a^No covariates were adjusted.

^b^Age, sex, and race/ethnicity were adjusted.

^c^Age was not adjusted in Model 2.

^d^Sex was not adjusted in Model 2.

^e^Race/ethnicity was not adjusted in Model 2.

CI, confidence interval, OR, odds ratio.

### Prevalence of metal implants and demographic factors

The results of weighted logistic regression models indicated that the metal implant prevalence differed by age and race/ethnicity, in which older individuals and White individuals showed a significantly higher prevalence of metal implants than younger individuals and non-White individuals, respectively. However, there were no significant differences in the prevalence of metal implants between men and women. The specific results are listed in Table 5.

**Table 5:** Weighted logistic regression analysis of factors associated with metal implants prevalence.

Demographic factor	Univariate		Multivariate	
	OR (95% CI)	*P* value	OR (95% CI)	*P* value
Age	1.05 (1.04, 1.06)	< 0.001	1.05 (1.04, 1.05)	< 0.001
Sex				
Men	Reference (1)	–	Reference (1)	–
Women	1.02 (0.89, 1.16)	0.804	0.97 (0.85, 1.10)	0.605
Race/ethnicity				
White	Reference (1)	–	Reference (1)	–
Non-White	0.57 (0.49, 0.66)	< 0.001	0.66 (0.56, 0.77)	< 0.001

CI, confidence interval, OR, odds ratio.

### Sensitivity analysis

In sensitivity analysis, this study assessed the prevalence of metal implants after excluding participants with missing data on demographic characteristics and medical history. After excluding participants with missing data on demographic characteristics and medical history, a total of 9,950 participants were included in the subsequent analysis. The prevalences of metal implants were similar compared with the results in the primary analysis (Table 3). The detailed results are listed in Supplementary Table S1.

## Discussion

To our knowledge, the present study is the first to evaluate metal implant prevalence among US adults aged 40 and older. The main findings of this study indicated that there was a high prevalence of metal implants among US adults aged 40 and older, with approximately one-third of those reporting the presence of metal implants inside the body. Moreover, this study observed that the prevalence of metal implants significantly increased from 2015 to March 2020, especially among older individuals, males, and White individuals. In addition, the present study demonstrated that the prevalence of metal implants differed by age and race/ethnicity, in which older individuals and White individuals showed a significantly higher prevalence of metal implants than younger individuals and non-White individuals, respectively.

This study observed a high prevalence of metal implants among US adults aged 40 and older, with 27.23% (95% CI: 23.61 to 30.85) in 2015–2016 and 31.53% (95% CI: 29.76 to 33.30) in 2017-March 2020. Moreover, the present study also observed a significant increase in metal implant prevalence from 2015 to March 2020 among US adults aged 40 and older. On the one hand, the aging US population might be a possible reason for this high prevalence. Several musculoskeletal degenerative disorders, such as osteoarthritis and osteoporosis, are strongly associated with increasing age^37–39^. Furthermore, the cumulative evidence indicates an increasing trend of these diseases, such as osteoarthritis and osteoporosis, in the US population^40–42^. Therefore, a significant increase in the number of older adults greatly enhances the probability that individuals receive surgery, such as THA or TKA, where metal implants are commonly used^10,11^. However, it should be noted that several non-orthopaedic conditions, such as vascular stents or cardiac implantable electronic devices, may also be an important cause of the high prevalence of metal implants because of the widespread usage of these materials mentioned above in clinical practice^43–45^. On the other hand, advancements in medical technology, in particular with regard to medical materials, might be another possible reason contributing to the high prevalence of metal implants. The development of more effective and safer metal implants plays an essential role in prolonging the service life of metal implants, improving the clinical outcomes of patients, and contributing to the increased use of metal implants in surgery^46,47^.

Interestingly, the present study observed a significant increase in metal implant prevalence among men but not women from 2015 to March 2020. The specific reasons for the sex differences remained unclear. There were several possible reasons as follows. First, it is important to note that certain medical conditions necessitating the use of metal implants may exhibit a higher prevalence among men. For instance, conditions such as hip arthritis or specific types of bone fractures are potentially more frequent in the male population, thereby resulting in an increased incidence of metal implant procedures in men^48^. Second, we speculated that the sex differences in orthopaedic injuries might be a possible reason because men tend to engage more in physically demanding activities or sports compared with women, which might lead to an increased risk of orthopaedic injuries^49–51^. The statistical significance of sex differences might help guide clinical strategies and public health policies. However, it was uncertain whether the statistical significance of sex differences had practical clinical implications. Therefore, additional studies are required to investigate whether the prevalence of metal implants differed by sex and potential cause that led to the gender differences in the prevalence of metal implants.

This study found race or ethnicity differences in the prevalence of metal implants, in which White individuals showed a significantly higher metal implant prevalence than non-White individuals. The differences in healthcare access and socioeconomic factors, such as healthcare, economic conditions, or cultural factors, which might result in disparities in the utilization of metal implants, could be responsible for the race or ethnicity differences in the prevalence of metal implants^52–54^. Numerous studies have highlighted the persistent disparities in healthcare access and utilization among different racial and ethnic groups in the US, which might lead to the higher prevalence of metal implants observed in whites compared with other races. For instance, a study examining the access to health care and preventive services between Asian Americans/Pacific Islanders (AAPIs) and Non-Hispanic Whites found that AAPIs were significantly less likely to have a personal healthcare provider and to receive various preventive services compared to their white counterparts^55^. Furthermore, the racial disparities in income and wealth level, which were reported in numerous previous studies^56,57^, might be a reason for the differences in the prevalence of metal implants. However, this study did not evaluate the healthcare access and socioeconomic factors among the participants included in the NHANES because this study mainly focused on describing the overall prevalence of metal implants and assessing the change in metal implant prevalence rather than the exploring the reasons for the differences in the prevalence of metal implants. Moreover, there was a large number of participants with missing data on healthcare access and socioeconomic factors, while there was no participant with missing data on age, sex, or race/ethnicity. Therefore, deeper analyses are needed to explore the reasons for the race or ethnicity differences in the prevalence of metal implants.

The main findings of this study have several implications for future research. To our knowledge, the present study is the first study to evaluate the prevalence of metal implants among US adults. The large population with metal implants and the increase in the metal implant prevalence suggest that this special population, those with metal implants inside the body, might be neglected, and there is a need to pay more attention to health care and health management for this special population. In addition, race or ethnicity differences in metal implant prevalence are observed in the present study. Although the specific reasons for these differences remain unknown, this topic is worth exploring further, which might be beneficial for developing pertinent policies and improving access to and the quality of healthcare services.

This study has some limitations, as follows. First, metal implant data were collected based on self-report, which might introduce recall bias and thus lead to inaccurate prevalence estimates. Future studies investigating the prevalence of metal implants could employ different methods to provide a more accurate estimation of the prevalence of metal implants, such as reliable medical records. Second, this study did not assess the prevalence of metal implants among subjects aged below 40 years, as the questionnaire on metal implants was not evaluated among those below 40 years, according to the information provided on the NHANES^58,59^. Therefore, future research could expand the scope of the investigation to get a comprehensive understanding of the actual usage of metal implants. Third, this study assessed metal implant prevalence after excluding a small number of individuals without definitive answers to the question on metal implants or with missing data on metal implants, which might influence the prevalence estimate, albeit at lower percentages (0.75%, 76 of 10,199). Fourth, this study did not estimate metal implant prevalence before 2015 or after March 2020 because the questionnaire on metal implants was not evaluated before NHANES 2015–2016, and data from the NHANES after March 2020 was not released. Therefore, a longer-term investigation seemed to be necessary and beneficial to evaluate the changing trends of the prevalence of metal implants. Fifth, this study did not assess the health insurance coverage of the study population. In the future, it is worth investigating the impact of health insurance coverage on metal implant utilization.

## Conclusions

The prevalence of metal implants among US adults aged 40 and older was high, with approximately one-third reporting the presence of metal implants inside the body, and the prevalence of metal implants significantly increased from 2015 to March 2020. Therefore, more attention needs to be paid to this special population, and it may be necessary to ensure accessibility and affordability and assess the potential long-term health impacts of metal implants, considering the increased prevalence of metal implants.

## Electronic supplementary material

Below is the link to the electronic supplementary material.

Supplementary Material 1

## Data Availability

The datasets obtained and analyzed in the present study are open access and can be downloaded at: https://wwwn.cdc.gov/Nchs/Nhanes/.
