# Supplementary material for: Prevalence of metal implants among US adults aged 40 years and older
Source: Sci Rep. 2025 Jan 2;15:584. doi: 10.1038/s41598-024-84340-0 (PMC11697384; doi:10.1038/s41598-024-84340-0)
Supplement: Supplementary file 1 — Supplementary Material 1 [file 41598_2024_84340_MOESM1_ESM.docx]

**Table S1 Sensitivity analysis for the prevalence of metal implants among US adults 40 years or older**

|  | **NHANES 2015-2016** | | **NHANES 2017-March 2020** | | |
| --- | --- | --- | --- | --- | --- |
| **Population** | **Unweighted sample size** | **Prevalence**  **% (95% CI)** | **Unweighted sample size** | | **Prevalence**  **% (95% CI)** |
| **Overall** | 3676 | 26.98 (23.39, 30.57) | 6274 | 31.30 (29.54, 33.07) | |
| **Age** |  |  |  |  | |
| 40-59 years | 1837 | 19.32 (15.45, 23.19) | 2964 | 21.16 (18.98, 23.34) | |
| 60 years and older | 1839 | 36.89 (32.49, 41.30) | 3310 | 43.09 (40.75, 45.44) | |
| **Sex** |  |  |  |  | |
| Men | 1759 | 25.37 (21.61, 29.13) | 3099 | 31.80 (29.09, 34.52) | |
| Women | 1917 | 28.41 (23.89, 32.93) | 3175 | 30.86 (28.78, 32.94) | |
| **Race/ethnicity** |  |  |  |  | |
| White | 1252 | 29.41 (25.16, 33.66) | 2338 | 35.72 (32.73, 38.72) | |
| Black | 789 | 20.73 (17.96, 23.50) | 1690 | 20.22 (17.93, 22.50) | |
| Mexican American | 609 | 19.12 (16.78, 21.47) | 632 | 23.21 (20.68, 25.75) | |
| Other | 1026 | 23.65 (18.33, 28.98) | 1614 | 23.42 (19.55, 27.29) | |

CI, confidence interval; NHANES, National Health and Nutrition Examination Survey.
